# Supplementary figures and images for: 16S-FASAS: an integrated pipeline for synthetic full-length 16S rRNA gene sequencing data analysis
Source: PeerJ. 2022 Sep 23;10:e14043. doi: 10.7717/peerj.14043 (PMC9511998; doi:10.7717/peerj.14043)

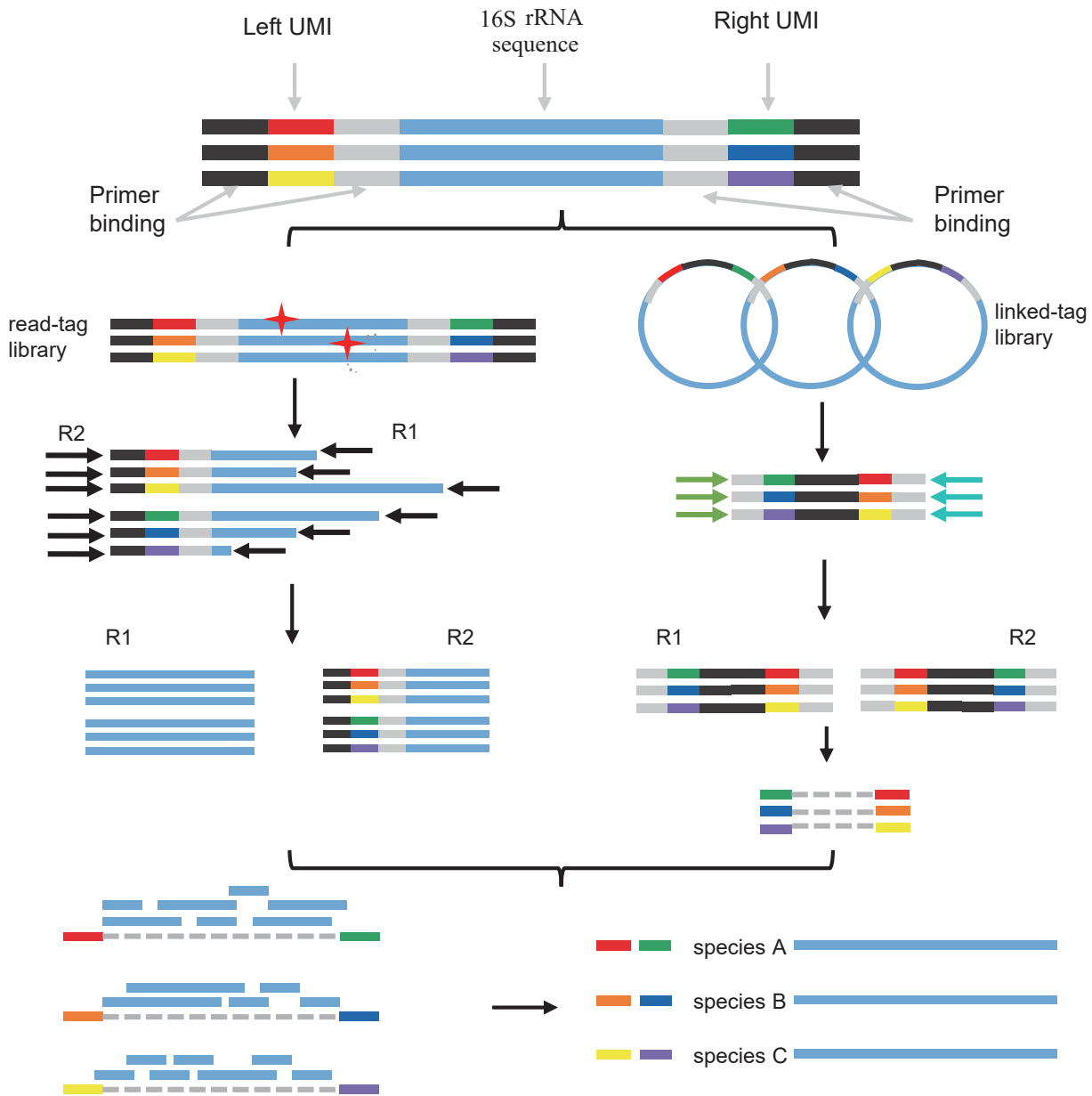

Supplement: Figure S1 — The cDNA molecules of full-length 16S rRNA are uniquely tagged with adaptors containing UMIs (unique molecular tags) and primer binding sites in both ends. The cDNA library are amplified by PCR and split into two parts. The linked-tag library is prepared by circularizing the cDNA molecules to make physical link between left and right UMIs. The read-tag library is prepared by random fragmenting the full-length 16S rRNA genes molecules. These two libraries are pooled and paired-end 150 bp sequenced using the Illumina MiSeq instrument. The direction orders of the paired-end reads are signed with arrow. The linked-tag pairs are used to bin all 16S rRNA gene fragment tag-reads originating from the same parent molecule. The read-tag reads with the same UMIs are used to re-create the parent full-length 16S rRNA gene molecules with a de novo assembly algorithm. [file peerj-10-14043-s001.pdf]
